# Society Meetings

**Published:** 1917-08

**Authors:** 


### SOCIETY MEETINGS

**Brief reports and announcements of meetings of societies of Western New York. and those of general scope, are requested from Secretaries. Copy should be on hand the fifteenth of the month. Full transactions will be published at cost of composition.**

---

**The Medical Society of the County of Erie** met June 18th, 1917, at 8:30 p. m., in the Buffalo Medical College, 24 High Street, Buffalo.

In the absence of the President and Vice-Presidents the Secretary, Dr. Franklin C. Gram, called the meeting to order and requested that a temporary Chairman be elected. On motion of Dr. Hopkins, Dr. DeLancy Rochester was elected Chairman pro tem.

Dr. Rochester thanked the Society and accepted the chairmanship for the evening.

The Secretary stated that the minutes of the previous regular meeting had been published in the Journal and on his motion the minutes were approved without being read.

The minutes of the Council meeting held May 22d were then read by the Secretary.

In these minutes a motion was made by the Council that $500 be taken from the sinking fund and invested in United States Liberty Bonds. Inasmuch as this was no longer possible, because bond selling had been closed and oversubscribed, the Society disapproved this motion of the Council.

The Council also recommended that the Society endow five beds in the Base Hospital and spend $250 for that purpose. This motion was approved by the Society.

The Council also recommended that the Society approve of the project of a Feeble-minded Colony at Akron, N. Y., and recommended the adoption of the following resolution:

Resolved, That the Medical Society of the County of Erie heartily endorse the action of the Vassar Club of Buffalo in establishing at Akron, N. Y., the Erie County Boys' Agricultural Colony under the direction of the Rome State Asylum.

That the Medical Society of the County of Erie recommend to the Board of Supervisors that it give moral and financial aid to this Colony by authorizing the Commissioner of Charities and Corrections to commit thereto proper individuals at proper rates; all for humanitarian as well as economical reasons.

That the President of the Medical Society of the County of Erie appoint a committee of five to inspect this Colony from time to time and to report to the Society at the annual meeting with recommendations.

That the Secretary of the Medical Society of the County of Erie be instructed to advise the Board of Supervisors, the Commissioner of Charities and Corrections, the Superintendent of the Rome State Asylum, and the Vassar Club of Buffalo of this action.

On motion of Dr. Wende the proposed resolutions offered by the Council were adopted by the Society.

The balance of the minutes of the Council meeting of May 22d, 1917, were then adopted.

The minutes of the special meeting of June 7th, 1917, were read by the Secretary and adopted.

Dr. Jacobs, Chairman of the Committee on Membership, then presented the applications of Dr. Ernest M. Watson, Electric Building, and Dr. Carl G. Frost, 1246 Hertel Avenue, Buffalo, N. Y.

The Secretary was instructed to cast the ballot of the Society for their election, which was duly done.

Dr. Jacobs also presented the application of Dr. J. H. Dewees for reinstatement in the Society. The Secretary was likewise instructed to cast the ballot of the Society for the reinstatement of Dr. Dewees.

Dr. Jacobs also presented the application of Dr. William P. Clothier for retired membership.

Dr. Bonnar made a verbal report of the Board of Censors, which was accepted.

Dr. Woodruff, Chairman of the Committee on Economics, reported progress.

Treasurer Lytle stated that the By-laws required the Treasurer to acquaint the Society with the names of all those in arrears with their dues at the June meeting. On motion of Dr. Bennett the reading of the names of those who were in arrears, as required in the By-laws, was omitted.

The scientific part of the program was then proceeded with.

The first was a lecture on ''Practical Methods for the Conservation of Vision,'' illustrated by lantern slides, by Dr. F. Park Lewis.

The next was a paper on ''Health Insurance and the Medical Profession, from the Financial and Administrative Point of View,'' by Dr. Albert T. Lytle.

The third was a paper on "The Demands upon the Civilian Physician in War Time," by Dr. Herbert A. Smith.

All of these papers were discussed and a vote of thanks given to those who presented them.

Adjournment followed.

FRANKLIN C. GRAM,
Secretary.

---

The Eighteenth Annual Meeting of the **Lake Keuka Medical and Surgical Association** was held in Keuka College Auditorium, Keuka Park, Thursday and Friday, July 5th and 6th, 1917.

Program:

1. Patriotic Address, by the President, I. H. Levy, M. D., Syracuse.
2. "Reminiscences of an Army Surgeon of the Civil War," John Van Duyn, M. D., Syracuse.
3. "Transfusion," E. Libman, M. D., New York.
4. "Injuries to the Brachial Plexus," Howard L. Prince, Rochester.
5. "The Sympathetic and Autonomic Systems in Relation to Subacute and Chronic Diseases," M. S. Woodbury, M. D., Clifton Springs.
6. "Are Gastric Symptoms Co-incident with Chronic Appendicitis and Gall Bladder Conditions Relieved by Surgical Interference?" H. C. Buswell, M. D., Buffalo.
7. "The Clinical Application of Some Special Urologic Methods of Diagnosis and Treatment," Leo Buerger, M. D., New York.
8. "Detection of Renal Disease in Routine Practice," A. A. Jones, M. D., Buffalo.
9. "Recent Advancement in the Study and Treatment of Kidney Disease," J. R. Williams, M. D., Rochester.
10. "General Streptococci Infection Through the Nasal Accessory Sinuses and Tonsils," T. H. Halstead, M. D., Syracuse.
11. "Informal Talk on Recto-Colonic Affections and Operations," illustrated by Motion Pictures, S. G. Gant, M. D., New York.
12. "Caesarian Section,"—A Continuation. W. M. Brown, M. D., Rochester.
13. "The Serum Treatment of Pneumonia," Augustus B. Wadsworth, M. D., Albany.
14. "Psycho-Analysis," A. A. Brill, M. D., New York.
15. "Experiences in a French Hospital," E. S. Van Duyn, M. D., Syracuse.

Moving pictures of maneuvers of the U. S. Medical Corps.

Officers for 1917: President, I. H. Levy, M. D., Syracuse, N. Y.; Vice-President, V. Lewis, M. D., Allegany, N. Y.; Secretary-Treasurer, E. C. Foster, M. D., Penn Yan, N. Y.; Recording Secretary, *W. W. Smith, M. D., Avoca, N. Y.; Committee of Arrangements, E. C. Foster, M. D., Penn Yan, N. Y.; P. L. Alden, M. D., Hammondsport, N. Y.; G. Howard Leader, M. D., Penn Yan, N. Y.; Bernard S. Strait, M. D., Penn Yan, N. Y.

*Deceased.

